# Supplementary material for: Crawling and Gliding: A Computational Model for Shape-Driven Cell Migration
Source: PLoS Comput Biol. 2015 Oct 21;11(10):e1004280. doi: 10.1371/journal.pcbi.1004280 (PMC4619082; doi:10.1371/journal.pcbi.1004280)
Supplement: S1 Code — (ZIP) [file pcbi.1004280.s012.zip › release/tst/doc/html/conrec_8cpp.html]

Tissue Simulation Toolkit: conrec.cpp File Reference


Tissue Simulation Toolkit
 0.1.4.1


- Main Page
- Namespaces
- Classes
- Files

- File List
- File Members

Macros |
Functions

conrec.cpp File Reference

`#include <stdio.h>`  
`#include <math.h>`  
`#include "graph.h"`  
`#include "conrec.h"`

Include dependency graph for conrec.cpp:

|  |  |
| --- | --- |
| Macros | |
| #define | xsect(p1, p2)   (h[p2]\*xh[p1]-h[p1]\*xh[p2])/(h[p2]-h[p1]) |
|  | |
| #define | ysect(p1, p2)   (h[p2]\*yh[p1]-h[p1]\*yh[p2])/(h[p2]-h[p1]) |
|  | |
| #define | min(x, y)   (x<y?x:y) |
|  | |
| #define | max(x, y)   (x>y?x:y) |
|  | |

|  |  |
| --- | --- |
| Functions | |
| int | conrec (double \*\*d, int ilb, int iub, int jlb, int jub, double \*x, double \*y, int nc, double \*z, Graphics \*g, int colour) |
|  | Paul Bourke's conrec algorithm to draw contour lines of PDE fields. More... |
|  | |

## Macro Definition Documentation

|  |  |  |  |
| --- | --- | --- | --- |
| #define max | ( |  | x, |
|  |  |  | y |
|  | ) |  | (x>y?x:y) |

Referenced by conrec(), PDE::ContourPlot(), and PDE::Max().

|  |  |  |  |
| --- | --- | --- | --- |
| #define min | ( |  | x, |
|  |  |  | y |
|  | ) |  | (x<y?x:y) |

Referenced by conrec(), PDE::ContourPlot(), and PDE::Min().

|  |  |  |  |
| --- | --- | --- | --- |
| #define xsect | ( |  | p1, |
|  |  |  | p2 |
|  | ) |  | (h[p2]\*xh[p1]-h[p1]\*xh[p2])/(h[p2]-h[p1]) |

Referenced by conrec().

|  |  |  |  |
| --- | --- | --- | --- |
| #define ysect | ( |  | p1, |
|  |  |  | p2 |
|  | ) |  | (h[p2]\*yh[p1]-h[p1]\*yh[p2])/(h[p2]-h[p1]) |

Referenced by conrec().

## Function Documentation

|  |  |  |  |
| --- | --- | --- | --- |
| int conrec | ( | double \*\* | *d*, |
|  |  | int | *ilb*, |
|  |  | int | *iub*, |
|  |  | int | *jlb*, |
|  |  | int | *jub*, |
|  |  | double \* | *x*, |
|  |  | double \* | *y*, |
|  |  | int | *nc*, |
|  |  | double \* | *z*, |
|  |  | Graphics \* | *g*, |
|  |  | int | *colour* = `1` |
|  | ) |  |  |

Paul Bourke's conrec algorithm to draw contour lines of PDE fields.

C-code Copyright (c) 1996-1997 Nicholas Yue.

References max, min, Graphics::Point(), xsect, and ysect.

Referenced by PDE::ContourPlot().


---

Generated on Thu Aug 14 2014 22:04:01 for Tissue Simulation Toolkit by  

 1.8.6
